# The peatland vegetation burning debate: keep scientific critique in perspective. A response to Brown *et al*. and Douglas *et al*.

**DOI:** 10.1098/rstb.2016.0434

**Published:** 2016-11-19

**Authors:** G. Matt Davies, Nicholas Kettridge, Cathelijne R. Stoof, Alan Gray, Rob Marrs, Davide Ascoli, Paulo M. Fernandes, Katherine A. Allen, Stefan H. Doerr, Gareth D. Clay, Julia McMorrow, Vigdis Vandvik

**Affiliations:** 1 School of Environment and Natural Resources, The Ohio State University, Kottman Hall, 2021 Coffey Road, Columbus, OH 43210, USA; 2 School of Geography, Earth and Environmental Sciences, University of Birmingham, Birmingham B31 2DX, UK; 3 Soil Geography and Landscape Group, Wageningen University, PO Box 47, 6700 AA Wageningen, The Netherlands; 4 NERC Centre for Ecology and Hydrology, Bush Estate, Penicuik, Edinburgh EH26 0QB, UK; 5 School of Environmental Sciences, University of Liverpool, Liverpool L69 3GP, UK; 6 Department of Agricultural, Forest and Food Sciences, University of Torino, Largo Paolo Braccini 2-10095, Grugliasco (TO), Italy; 7 Centro de Investigação e de Tecnologias Agro-Ambientais e Biológicas, Universidade de Tras-os-Montes e Alto Douro, Vila Real, Portugal; 8 Department of Geography, Swansea University, Swansea, UK; 9 School of Environment, Education and Development, The University of Manchester, Manchester M13 9PL, UK; 10 Department of Biology, University of Bergen, Postboks 7803, 5020 Bergen, Norway

We are glad that Brown *et al*. [1] and Douglas *et al*. [2] agree that there is a need to move forward in the debate regarding the use of fire as a management tool in the UK uplands and appreciate their robust responses to some of the issues we identified. We may not agree, but discussing these problems and balancing the current debate from an ecological viewpoint is important. Our recent paper [3] contained a critique of certain aspects of two recent papers they published [4] and [5]. We believe this critique was important, because we believe the interpretations they provided sometimes lacked adequate engagement with existing research on peatland fire ecology, had the potential for damaging misinterpretation, and occasionally appeared to have an unintentional lack of balance. In the case of Brown *et al*. [4], this concern was exacerbated by the fact it was a review paper and such publications aim to provide an authoritative overview of knowledge in a certain area. We believe there were several respects in which that standard was not met. We also critiqued media outreach and coverage associated with their papers and, in the case of Brown *et al*. [3], the publication protocol associated with a research report they issued [6]. Here, we briefly address Brown *et al*. and Douglas *et al*.'s main concerns regarding our recent paper.

Many of the issues raised by Brown *et al*. are associated with a particular interpretation of our language. While our original paper emphasized the importance of precision of language, for reasons of brevity, we are not able to engage with all such criticisms here and do not feel it is productive to get into a prolonged debate about how we or they may have phrased things better. We will simply state that where we offered a critique of their tone or interpretation, we did so having carefully read their research, corresponded with co-workers about it, and then raised specific concerns about how it could be perceived. We remain willing to clarify our concerns in correspondence should the authors wish.

Brown *et al*. were concerned over our criticism of their statement that ‘burning is considered particularly detrimental to peat-forming *Sphagnum* species'. As they acknowledge in their comment, there was a single reference provided in their review paper with regard to *Sphagnum* sensitivity to managed fires [7]. We referred to this as an unpublished report, because free availability and an ISBN does not equate to formal scientific publication, particularly where there is no indication of peer review. Following the citation trail in Grant *et al*. [7] for their initial assertion regarding *Sphagnum* [4] reveals three references. Two are non-technical publications that predate much research on peatland fire ecology in the UK and elsewhere, and none are peer-reviewed [8–10]. Burning involves biomass combustion, so we cannot dispute that when fires occur in peatlands *Sphagnum* biomass could be lost. However, during management fires, this is unlikely owing to *Sphagnum's* very high moisture content. Smouldering combustion might occur during severe fires in periods of drought. Although there is palaeoecological data that provides circumstantial evidence that some *Sphagnum* species may be sensitive to land-use intensification and burning [11,12], there is abundant evidence that *Sphagnum* can regenerate following burning [13,14] and that some species may be favoured by managed fire [15]. There is a lack of evidence for temporal and spatial effects of fire on *Sphagnum* in general and severe wildfire effects should not be confounded with the outcomes of prescribed burns. It remains unclear why a managed (thus low severity) fire should be particularly detrimental to *Sphagnum,* because it has the ability to regenerate from stems many centimetres below the capitulum [16] and fire-induced belowground heating is very limited [17]. We hope Brown *et al*. will understand that our concern was that they were propagating a highly generalized supposition (‘Burning is considered particularly detrimental to peat-forming *Sphagnum* species') which remains largely unsupported in the scientific literature.

Brown *et al*. criticize us for disagreeing with their concerns about the controllability of prescribed fires, in particular the potential for combustion of moss and litter layers. It would obviously be ludicrous to state that consumption of such layers is physically impossible. Rather, we pointed out that available experimental evidence suggests that this can be minimized by burning under appropriate fuel moisture conditions [18]. We also felt it was inaccurate for them to conflate the difficulty of fire control (i.e. fire intensity) with consumption/heating of the moss and litter layer (i.e. fire severity). We refer readers to Keeley [19] for further discussion of this issue.

Regarding our critique of the release, and press coverage, of the non-peer-reviewed EMBER report [6], we acknowledged, in our paper, that many of the results have since been published in peer-reviewed journals. In contrast to what they state in their comment, we did not suggest that the results of their recent review [4] influenced media coverage of their report—all entries in our table 1 are referenced as being associated with the EMBER report [6]. In our paper, we made no criticism of the basic science in the EMBER report. We would argue that if scientists need to issue potentially controversial research reports to, for example, satisfy funders, they should develop and report their codes of best practice. Wherever possible such reports should be peer-reviewed. Research reports should describe methodologies in full either in the report itself or in a technical supplement as there is no guarantee all results will eventually be accepted by scientific journals.

With regard to criticism of our perception study by Brown *et al*. and Douglas *et al*., we clearly stated that the participants were a mixture of senior (final-year) undergraduates and graduate students studying ecosystem restoration. Undergraduate students were predominantly majors in environmental science or forestry, fisheries and wildlife. We did not collect data on the participants' gender, nor did we collect data on their race, age, marital status, sexual orientation or socio-economic background. Readers were assigned to random groups and were asked to reach a consensus which they reported for the group as a whole. This approach does not lend itself to formal statistical analysis but is a legitimate qualitative approach to a socio-scientific question [20]. Our reporting of the study is fully appropriate given its scope—we are happy to provide the detailed materials and methods we used should anyone wish to duplicate it, we would be interested to hear what results they get.

Douglas *et al*. criticize us for inconsistency in our description of the seasonal distribution of wildfires in the UK. We are happy to clarify any misunderstanding. If readers examine the reference in question [21] they will see that wildfires display a bimodal seasonal distribution. The larger peak does, indeed, occur in early spring and may be associated with both fire weather conditions and the prevalence of ignitions (presumably at least in part from escaped managed burns). A second large peak occurs in summer and is primarily associated with warm dry weather. Management burns are legally constrained between October and mid-April (exact dates vary by location and elevation). A paper on the seasonal variation in wildfire activity in Scotland is currently under a review.

A number of the authors here have previously outlined their concerns regarding Douglas *et al*.'s interpretation of their MODIS data [22] and why the balance of evidence suggests the fires they detected are likely to be large wildfires rather than to be much smaller managed burns. We are very aware of the challenges of using and interpreting MODIS active fire data both in general and in a UK context. We have previously corresponded with one of the authors of Douglas *et al*. [2] on this subject and are happy to continue doing so if we can provide useful insights from related studies a number of us have undertaken [23,24]. We will not repeat our critique here except to again note that (i) the number of ‘managed fires' Douglas *et al*. [5] reported from their MODIS data is rather small compared with the number of wildfires reported by the Fire and Rescue Services, let alone the number of managed fires burnt by managers; and (ii) the probability of MODIS detection for most managed fires will be less than 50% even in perfect viewing conditions.

With regard to our estimation of fire rotations, criticized in Douglas *et al*. [2], we clearly stated that we used the data available from Douglas *et al*. [5] to make a rough estimate of mean fire rotation (defined as per [25]) and annual area burnt. Confusion may have been caused as we later stated ‘…average fire-return interval of 147 years…’ when we should have referred to a ‘mean fire rotation’. The values we provided are not misleading; they should certainly not be taken as absolute but usefully illustrate the significant variation in fire regimes across the UK. Douglas *et al*. have at their disposal an extremely valuable dataset that could be used to interpret variation in fire regimes. We would encourage them to consider Romme [25] and we look forward to them developing their analysis further than we were able to based on the simple summary statistics reported in their original paper.

Our paper outlined why we believe that wholesale changes in management should be considered carefully as we currently have little evidence of what the environmental consequences of actions such as the total cessation of burning would be. Douglas *et al*. are correct that there is growing pressure being placed on the use of managed burning by water companies (for instance burn bans on the significant areas of peatland they own) owing to concern regarding dissolved organic carbon, DOC). As we outlined in our paper, there is currently no clear consensus that existing evidence suggests managed burning alone drives increased DOC concentrations or has negative consequences for C storage (e.g. different responses at different scales have been reported). Many existing studies suffer from complexities introduced by interacting disturbances, including fire, grazing and drainage. We demonstrated that assessments of habitat condition are overly simplistic and do not account for the ecological role of fire in peatlands. Douglas *et al*. advocate a precautionary response to these challenges; this might be appropriate in some locations where services such as drinking water are of critical importance. Given existing uncertainty there is no guarantee that such changes will provide the desired benefits. A passive Adaptive Management approach [26] is therefore required. We have not argued that burning should be used everywhere, and nor do we suggest the *status quo* associated with grouse moor management (such as generalizations about highly variable practices are possible) is appropriate everywhere either. We would point out that wildfire control and fuel reduction treatments may be important in areas where burning ceases. Although this requires further study, the results reported in Allen *et al*. [27] highlight potential benefits. Prescribed fire is a flexible tool that can be used in a targeted fashion to ensure that landscapes at large experience a lengthened fire return interval. Upland ecosystems support a diverse range of ecosystem services, important plant communities and wildlife populations as well as agricultural and game production. Managing for a single ecosystem service, be it red grouse, drinking water or carbon, is unlikely to lead to holistically managed, diverse upland landscapes. We advocate an Adaptive, evidence-based approach where decision-making on land-use priorities takes place locally, in a participatory manner and in which clear objectives and on-going monitoring are used to facilitate adaptation. Achieving ecological management objectives will require a range of tools, which, depending on the local circumstances, may include burning.

In their conclusion, Brown *et al*. suggest we have added to the partisan tone of the debate. We are content that our paper has instead rebalanced the conversation regarding peatland fire management and subjected it to a rigorous assessment from the perspective of fire ecology. Specific criticisms we have made while demonstrating our points should be kept in perspective—science proceeds by debate and the formulation of questions or hypotheses, followed by evidence gathering to address the questions and then further debate. The complex questions associated with the effects of fire in peatlands are best addressed by appreciating perspectives and expertise from a range of different disciplines. We hope Brown *et al*. and Douglas *et al*. will be willing to reflect on and appreciate ours. We retain admiration for much of their work aside from our disagreements here.
